# Trends in Prevalence of Clonorchiasis among Patients in Heilongjiang Province, Northeast China (2009–2012): Implications for Monitoring and Control 

**DOI:** 10.1371/journal.pone.0080173

**Published:** 2013-11-19

**Authors:** Su Han, Xiaoli Zhang, Rui Chen, Jingshan Wen, Yihong Li, Jing Shu, Hong Ling, Fengmin Zhang

**Affiliations:** 1 Department of Microbiology and Parasitology, The Heilongjiang Key Laboratory of Immunity and Infection, Pathogenic Biology, Harbin Medical University, Harbin, People’s Republic of China; 2 Department of Spine Surgery, Second Affiliated Hospital of Harbin Medical University, Harbin, People’s Republic of China; 3 Key Laboratory of Bio-Pharmaceutical, Harbin Medical University, Ministry of Education, Harbin, People’s Republic of China; University of Illinois at Chicago, United States of America

## Abstract

**Background:**

Clonorchiasis is an important zoonotic parasitic disease worldwide. Past estimates showed the prevalence increased based on studies undertaken ten years or more ago. However, control strategies, changing ecology and migration may have resulted to changes in the prevalence of clonorchiasis. The purpose of the present study was to analysis the prevalence and epidemiological characterisation of clonorchiasis in Heilongjiang Province, Northeast China.

**Methodology/Principal Findings:**

A total of 4951 clinically suspected outpatients were examined from January 2009 to December 2012. Overall prevalence of clonorchiasis was 25.93% (1284/4951) by the combination strategy of the Kato-Katz technique (KK) and enzyme-linked immunosorbent assay (ELISA), with a significant increase from 22.53% in 2009 to 34.25% in 2012. Apart from *Daxinganling* city, clonorchiasis was reported throughout Heilongjiang Province and mainly along the Songhua River and Nen River basin, with an increased annual prevalence. The annual prevalence in men increased significantly in 2012 and was higher than that in women over 4 years. A similar pattern was seen for the annual infection rate in rural and urban areas. Farm labourers accounted for the majority of cases (65.93%), with a higher prevalence than in other occupations. Consumption of freshwater fish was considered the strongest risk factor of clonorchiasis. The infection rates in the 40–49 and 50–59 years age groups showed a significant increasing trend in 2012. Cases of re-infection were common.

**Conclusions/Significance:**

The present study revealed that clonorchiasis remained widespread and prevalent in Heilongjiang Province. An integrated control programme is urgently needed to reduce the public health impact of clonorchiasis in this endemic area.

## Introduction

Clonorchiasis, caused by *Clonorchis sinensis* (*C. sinensis*), is an important foodborne zoonotic disease, with an estimated 35 million people infected worldwide [1,2]. Humans and mammals can be infected by consuming raw or inadequately cooked freshwater fish containing the infective metacercariae. Infection by *C. sinensis* induces several pathological changes including chronic inflammation, periductal fibrosis, and epithelial desquamation in the bile duct. Hepatobiliary diseases such as cholangitis, hepatic fibrosis, and even cholangiocarcinoma and liver cancer can be associated with *C. sinensis* infection [3–5]. Recently, *C. sinensis* has been classified as a type 1 carcinogen to humans and among the neglected tropical diseases [6,7]. 

 Clonorchiasis has been an important public health problem in China, especially in Heilongjiang Province [8]. According to national sampling surveys in China, the prevalence of clonorchiasis increased by 75% from 1990 to 2003, and Heilongjiang Province had been confirmed as an endemic focus [4,9]. However, this latest investigation had been published for ten years. Furthermore, a few recent large-scale studies have focused on prevalence of *C. sinensis* in this endemic region. It is imperative to evaluate further the epidemiological factors and prevalence due to changes in socioeconomic conditions, human behaviour and environmental factors. 

The present cross-sectional investigation from January 2009 to December 2012 was performed on outpatients using a combination of the Kato–Katz (KK) method and ELISA. The aim of this study was to assess the change in prevalence and epidemiological characterisation of clonorchiasis in Heilongjiang Province, Northeast China over time, which will provide a basis for effective disease control planning and monitoring.

## Materials and Methods

### Ethics statement

The procedures of sample collection and use were approval by the Ethics Committees of Harbin Medical University. The objectives, procedures and potential risks were explained to all participants. Written informed consents were obtained from all adult participants and the parents or legal guardians of children. Individuals with positive fecal examination results were treated with a single 40 mg/kg dose of praziquantel three times a day. 

### Study area and participants

This study was conducted from January 2009 to December 2012 in Heilongjiang Province. The province is situated in the northeast of China, stretching from 121° 11' to 135° 05 'E longitude and 43° 25' to 53° 33' N latitude. The winters are cold and snowy, and the summers are hot and rainy. The province has an area of approximately half a million square kilometers, with about 38 million population. It owns 13 administrative regions, with the city of Harbin as its capital. In terms of ecology, 37% of the province is characterized by plains. 

Based on the national standardized diagnostic criteria for suspended patients published by the Ministry of Health of China (WS309-2009), a total of 4951 outpatients (male 3635 and female 1316) raging in age from 5 to 86 years old were collected who referred to the Department of Parasitology, Harbin Medical University for parasite examination. 

### Questionnaire

An individual questionnaire was used to obtain information on socio-demographic variables, including name, age, sex, occupation, residence, history of eating raw freshwater fish and/or shellfish, as well as any previous history of the disease treatment recorded etc. 

### Immunological tests

Blood samples from all participants were collected and tested by well trained technicians using an indirect ELISA diagnostic kit from Shenzhen Combined Biotech Co. Ltd., China. The technicians were unaware of the subject’s medical status. The incubation procedure, washing steps and detection steps were carried out according to instructions supplied by the manufacturer. Absorbance was read at 450 nm zeroed by the reagent blank wells. For each run, positive and negative control sera were measured simultaneously. A positive result was defined as an optical density (OD) value greater than 2.1 times the OD value of the negative control serum provided by the kit.

### Stool examination

The fecal samples were collected from each patient (1–3 specimens per patient) and detected *C. sinensis* eggs by the modified kato thick smear method (Kato-Katz technique) [10]. Briefly, the procedure of the method was as follows: each fecal sample was sieved through a fine screen and filled into a hole of a plastic template (41.7mg); the calibrated stool in slide was covered with cellophane soaked in glycerol and malachite green, then pressed against a hard surface so that the stool can spread evenly; after clarification overnight, triple slides were made from each stool specimen. Slides were read 1-12h after their initial preparation by two experienced technicians in a blinded manner. To maximize the diagnostic accuracy, the seropositive and egg-negative cases were re-examined by repeated egg counts and/or the number of KK slides. Patients were considered positive when at least one stool sample was found eggs of *C. sinensis*, which was considered to be the standard. The prevalence of *C. sinesis* infection was referred to as egg-positive rate.

### Statistical analysis

SPSS (version 10.0 software for windows; Chicago, IL, USA ) was used for analyzing the date. "Number positive" in Table 1 through 3 was referred to the number of egg-positive individuals. The chi-square test was used to evaluate the assessment between qualitative variable to check for statistical differences. Unconditional multivariate analysis was used to calculate odds ratios (Ors) and corresponding 95% confidence intervals (CIs) of being *C. sinensis* egg positive according to various characteristics. P<0.05 was regarded as statistically significant.

**Table 1:** Prevalence of clonorchiasis among Patients in Heilongjiang Province, China.

Year	No. examined	No. positive	Prevalence (%)
2009	870	196	22.53
2010	1489	317	21.29
2011	1316	334	25.38
2012	1276	437	34.25*
Total	4951	1284	25.93

* P<0.001, significantly different from 2009 vs 2012, 2010 vs 2012, 2011 vs 2012.

## Results

### Annual trends of clonorchiasis prevalence

Within the last 4 years (2009–2012) a total of 4951 were requested for *C. sinensis* diagnosis and 1284 (25.93%) microscopically confirmed clonorchiasis cases. There was a fluctuating trend of clonorchiasis within the last 4 years with lower prevalence (21.29%; 317/1489) in 2010 and higher (34.25%; 437/1276) in 2012. In addition, the prevalence of clonorchiasis in 2012 increased significantly (*P*<0.05) (Table 1).

### Geographical distribution of clonorchiasis

**Figure 1:**
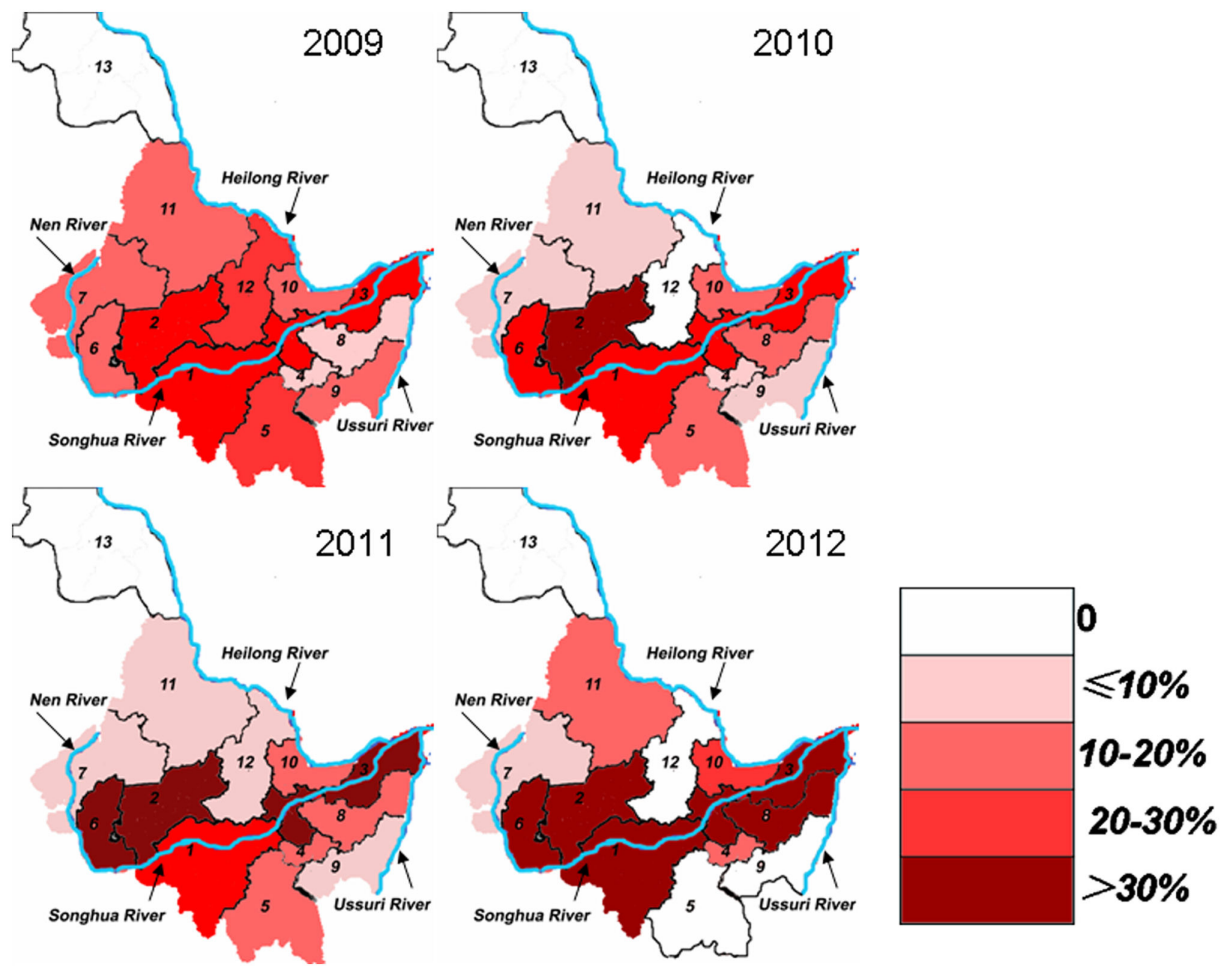
Geographical distribution of clonorchiasis among Patients in Heilongjiang Province.

Clonorchiasis geographical distribution in various sites was shown in Table 2 and Figure 1. In the Songhua River and Nen River basin, the prevalence rate (35.67%; 412/1155) in 2012 was considerably higher than that in the past 3 years (*P*<0.05). The prevalence in *Harbin*, *Daqing* and *Jiamusi* cities remained relatively stable in 2009–2011, and increased significantly in 2012 (*P*<0.05). Although not significant, the prevalence rates in *Suihua* showed an increasing trend, *Qiqihar* showed a declining trend, and *Qitaihe* and *Mudanjiang* fluctuated. 

**Table 2:** Geographical distribution of clonorchiasis among Patients in Heilongjiang Province.

Locality ^a^	2009		2010		2011		2012	
	%	No./N	%	No./N	%	No./N	%	No./N
Songhua River and Nen River	23.60	181/767	22.41	298/1330	26.60‡	315/1184	35.67^*^	412/1155
(1) Harbin	24.58	117/476	21.93	191/871	25.83	203/786	34.53^*^	280/811
(2) Suihua	22.43	24/107	30.77	44/143	30.43	42/138	36.21	42/116
(3) Jiamusi	26.51	22/83	25.78	33/128	30.16	38/126	44.34^†^	47/106
(4) Qitaihe	10.00	1/10	8.33	1/12	16.67	4/24	20.00	1/5
(5) Mudanjiang	30.00	6/20	11.11	4/36	16.67	2/12	0.00	0/6
(6) Daqing	14.81	8/54	22.12	23/104	31.25	25/80	45.05^†^	41/91
(7) Qiqihar	17.65	3/17	5.56	2/36	5.56	1/18	5.00	1/20
Ussuri River	11.11	4/36	14.06	9/64	17.86	10/56	22.22	10/45
(8) Shuangyashan	8.33	2/24	16.67	7/42	20.00	9/45	32.26	10/31
(9) Jixi	16.67	2/12	9.09	2/22	9.09	1/11	0.00	0/14
Heilong River	16.42	11/67	10.53	10/95	11.84	9/76	19.74	15/76
(10) Hegang	18.52	5/27	15.09	8/53	25.93	7/27	28.89	13/45
(11) Heihe	16.00	4/25	7.69	2/26	5.00	1/20	13.33	2/15
(12) Yichun	25.00	2/8	0.00	0/4	4.17	1/24	0.00	0/9
(13)Daxinganling area	0.00	0/7	0.00	0/12	0.00	0/5	0.00	0/7

^a^ locality from the province in Figure 1. No.: Number positive; N: Number examined; ^*^ p<0.05 2012 vs 2009, 2012 vs 2010, 2012 vs 2011; † p<0.05 2012 vs 2009, 2012 vs 2010; ‡p<0.05, 2010 vs 2011

In the Ussuri River basins, the prevalence rate increased year by year from 2009 (11.11%; 4/36) to 2012 (22.22%; 10/45). The prevalence rates in *Shuangyashan* showed an upward tendency, and *Jixi* showed a downward trend within 4 years. 

In the Heilong River basins, there was also a fluctuating trend of clonorchiasis within the last 4 years with lower prevalence (10.53%; 10/95) in 2010 and higher (19.74%; 15/76) in 2012. The prevalence of *Hegang*, *Heihe* and *Yichun* also showed fluctuated. However, no *C. sinensis* cases were detected in *Daxinganling region.*

The overall prevalence of the Songhua River and Nen River basin during the past 4 years was 27.19% (1206/4436), which was higher than in Heilong River (14.33%; 45/314) and Ussuri River (16.42%; 33/201) (*P*<0.05). 

### Age distribution of clonorchiasis in Heilongjiang Province

**Figure 2:**
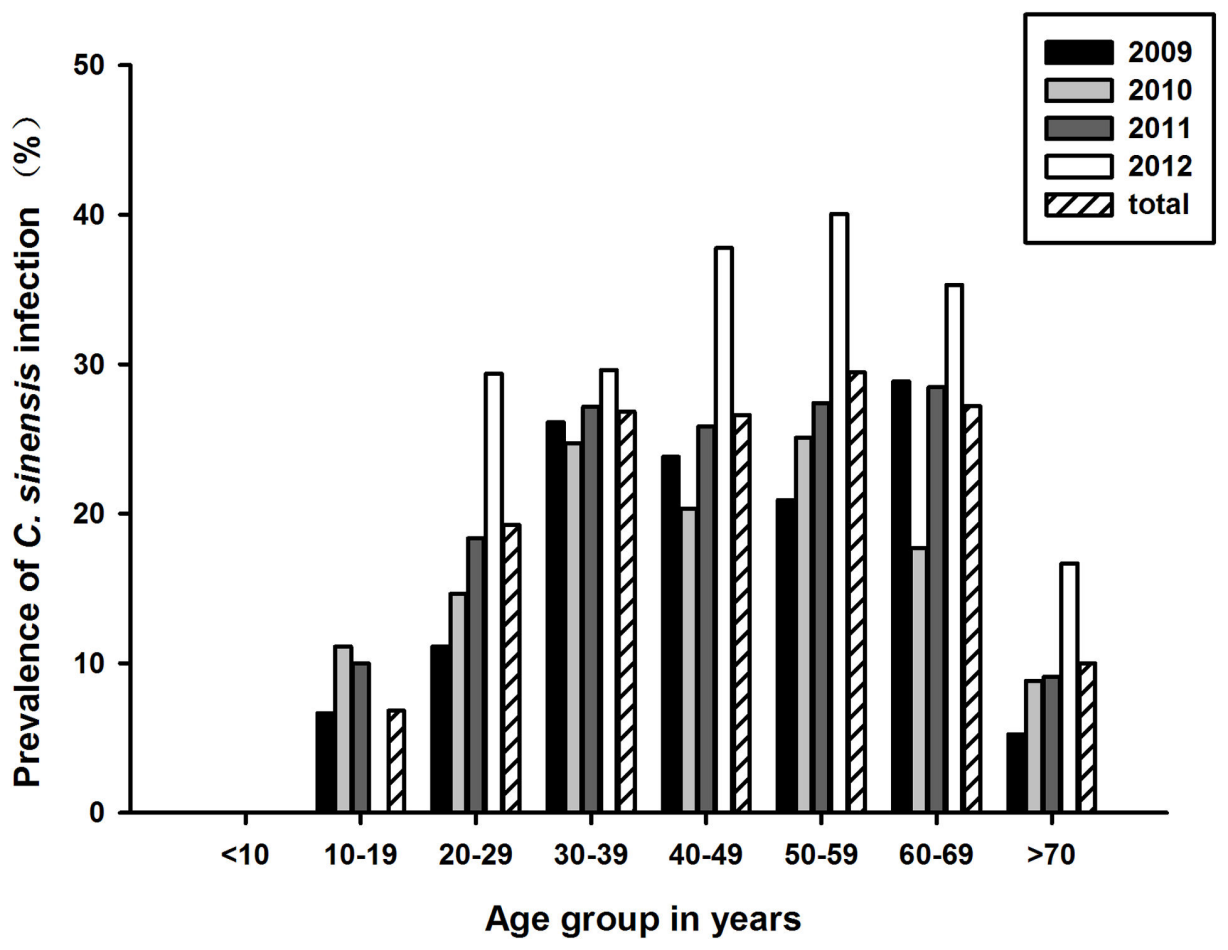
Prevalence of clonorchiasis in different age groups of Patients.

Clonorchiasis was reported in all age groups in the area except for <10 years group and the prevalence increased with age. The youngest person infected was 10 years old and the oldest was 82 years old. In 2009 and 2011 years, the prevalence rate reached a peak at 60–69 years with 28.85% and 28.46%, respectively. While in 2010 and 2012 years, it was higher in the 50–59 age group with a prevalence rate of 25.08% and 40.06%, respectively (Figure 2).

### Epidemiological characteristics of clonorchiasis

In the last four years, males showed a significantly higher (*P*<0.001) egg-positive rate (28.67%) than females (18.39%). The rate of infection in rural areas (30.48%) was significantly higher than that found in the urban (14.11%) (*P*<0.001). The majority of human cases (82.00%; 4060/4951) had eaten raw freshwater fish and/or shellfish. In 4060 patients with a past history of ingestion, the infection rate was 31.13%. Raw freshwater fish consumption was strongly associated with clonorchiasis (OR=16.30; 95% CI=10.38–25.58). With regard to occupations, the farm labourers accounted for the majority 65.93% (3264/4951), with a higher prevalence (30.88%), followed by civil servants with the prevalence rate (18.29%), significantly greater (P<0.001) than the proportion of any other occupation (8.31%). There was no difference of prevalence in the history of treatment and the different each season, respectively. (Table 3). 

**Table 3:** Univariate analysis of factors associated with clonorchiasis among Patients in Heilongjiang Province.

Variable	Subcategory	Positive n (%)	Negative n (%)	*β*	*S_X_*	Wald χ^2^	*P*
Total		1284 (25.93)	3667 (74.07)				
Gender	Male	1042 (28.67)	2593 (71.33)				ref.
	Female	242 (18.39)	1074 (81.61)	-0.5785	0.0801	52.2263	<0.001
Environment	Rural	1090 (30.48)	2486 (69.52)				ref.
	Urban	194 (14.11)	1181 (85.89)	-0.9817	0.0856	131.6520	<0.001
History of treatment	No	1112 (25.96)	3172 (74.04)				ref.
	Yes	172 (25.79)	495 (74.21)	-0.0089	0.0951	0.0087	0.9259
Habit of eating raw fish	no	20 (2.24)	871 (97.76)				ref.
	Yes	1264 (31.13)	2796 (68.87)	2.9799	0.2287	169.8107	<0.001
Season	Spring	277 (26.26)	778 (73.74)				ref.
	Summer	396 (26.67)	1089 (73.33)	0.0211	0.0913	0.0534	0.8172
	Autumn	322 (25.64)	934 (74.36)	-0.0322	0.0952	0.1144	0.7352
	Winter	289 (25.02)	866 (74.98)	-0.0647	0.0975	0.4408	0.5067
Occupation	Other	27 (8.31)	298 (91.69)				ref.
	Farmer	1008 (30.88)	2256 (69.12)	1.5956	0.2045	60.8691	<0.001
	Civil servants	248 (18.29)	1113 (81.71)	0.9039	0.2129	18.0330	<0.001

Multivariate logistic regression analysis using forward stepwise confirmed that sex, eating raw fish, location, and occupation were closely related to clonorchiasis (Table 4). 

**Table 4:** Multivariate logistic regression analysis of factors associated with clonorchiasis among Patients in Heilongjiang Province.

Variable	*β*	*S_X_*	Wald χ^2^	*P*	OR (95%CI)
Intercept	-4.6803	0.3082	230.6364	<0.001	—
Gender	-0.3494	0.0848	16.9748	<0.001	0.71 (0.60-0.83)
Habit of eating raw fish	2.7910	0.2300	147.2322	<0.001	16.30 (10.38-25.58)
Environment	-0.6111	0.1057	33.4441	<0.001	0.54 (0.44-0.67)
Occupation					
Farmer / Other	1.4100	0.2097	45.2066	<0.001	4.10 (2.72-6.18)
Civil servants / Other	1.1933	0.2186	29.7997	<0.001	3.30 (2.15-5.06)

### Change of epidemiological characteristics in different patient populations

**Figure 3:**
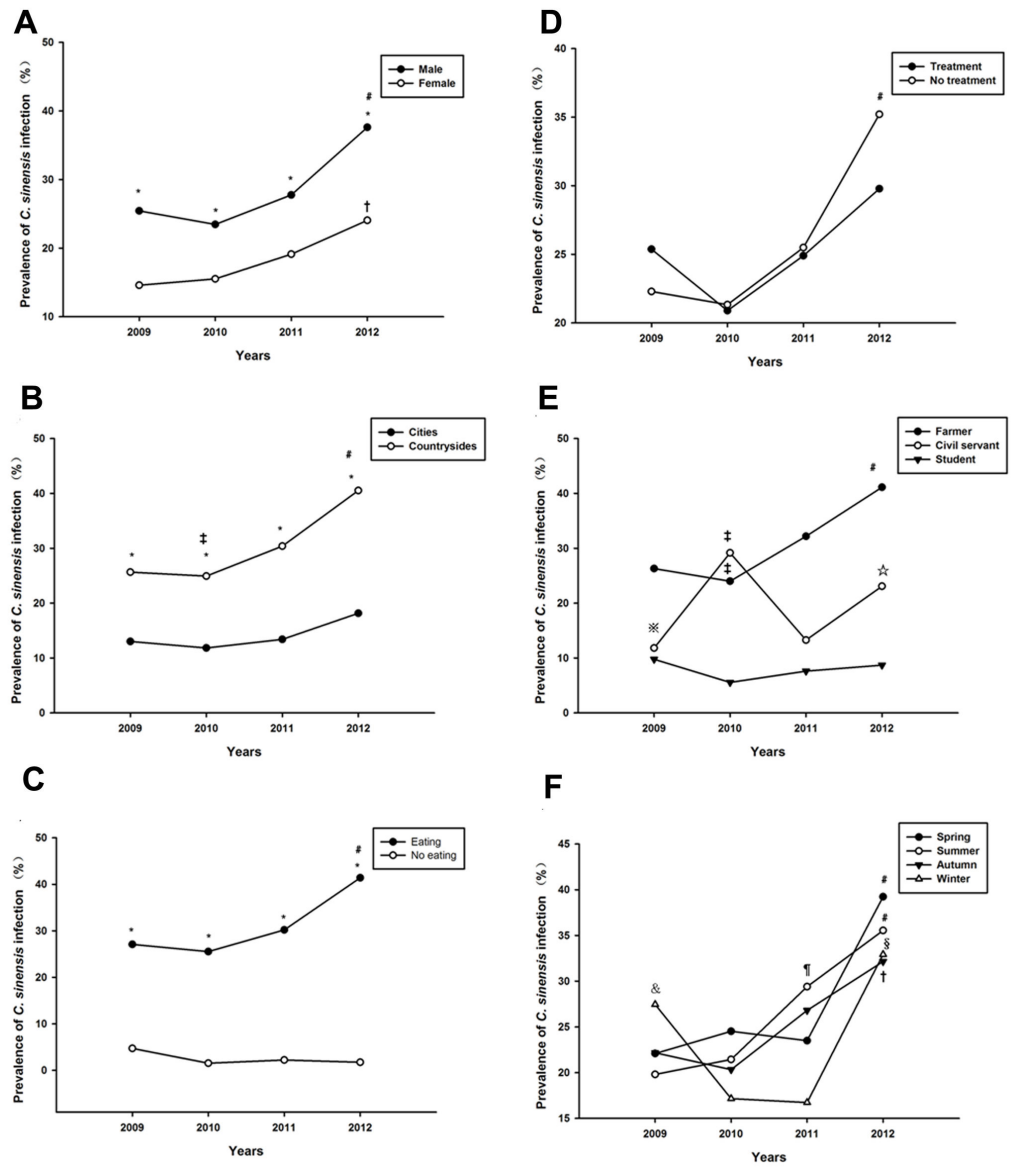
Prevalence of clonorchiasis in different patient populations. A. Gender. B. History of treatment. C. Environment. D. Occupation. E. Habit of eating raw fish. F. Season. *P<0.05, compared at the same time point of prevalence of *Clonorchis sinensis* infection in different patient populations. ﹟P<0.05, 2009 vs 2012, 2010 vs 2012, 2011 vs 2012; †p<0.05, 2009 vs 2012, 2010 vs 2012; § p<0.05, 2010 vs 2012, 2011 vs 2012; ☆p<0.05, 2011 vs 2012; ¶ p<0.05, 2009vs 2011, 2010 vs 2011; ‡p<0.05, 2010 vs 2011; ＆p<0.05, 2010vs 2009, 2011 vs 2009; ※p<0.05, 2010vs 2009, 2012vs 2009.

Figure 3 showed the changes in epidemiological characteristics of clonorchiasis in different patient populations. For the period 2009–2012, the annual prevalence in men increased significantly, and was higher than that in women every year (*P*<0.05) (Figure 3 A). A similar pattern was seen for the annual infection rate in surrounding and habit of eating raw fish, respectively. The annual prevalence in rural increased significantly, and was higher than that in urban areas every year (*P*<0.05) (Figure 3 B). Figure 3 C also showed that the infection rates of human cases had eaten raw freshwater fish owed a higher prevalence than that without habit. 

With regard to other factors including history of treatment, occupation and season, the annual infection rates revealed fluctuated irregularly (Figure 3 D, E, F). 

## Discussion

The findings from this study proved that the prevalence of clonorchiasis, as a representative food-borne parasitic disease, was still a serious public health problem in Heilongjiang Province. The prevalence of *C. sinensis* was 1.2% in 1988 [1,11]. However, the mean prevalence was 9.5% in studies conducted from 1988 to 2002, with 67 666 people from 21 different sites being subjected to fecal examination [1]. In the present study, 1284/4951 (25.93%) individuals were infected with *C. sinensis* over 4 years. The infection rate was lower than that of 31.6% in Hunan Province [12], but higher compared to that in other studies on the general population in Korea (16%) and Vietnam (17.2%) [13,14]. Different infection rates are related to many factors, including the sensitivity and specificity of diagnostic methods, the size and structure of specimens, the study subjects, and study areas. In addition, socioeconomic, demographic, biological and environmental factors are related to the transmission dynamics of *C. sinensis* [15].

From 2009 to 2012, the prevalence of clonorchiasis increased from 22.53% to 34.25%. Although other diseases such as schistosomiasis and soil-transmitted helminthiasis have declined significantly in China as a result of long-term treatment and control efforts [16,17], the prevalence of clonorchiasis has been consistently high. The change in epidemiological and socioeconomic factors, including more migration, more opportunities for eating, rapid growth of aquaculture, and lack of self-protection awareness has contributed to the high infection rates [18]. This present study suggests that our future parasite prevention and control work should focus on *C. sinensis* in this province.

In terms of geographical distribution, our data showed that clonorchiasis was widely prevalent in Heilongjiang Province, and mainly along the mainstream and tributaries of the Songhua River and its northern source Nen River. The reason might be that Songhua River and Nen River are important sites for freshwater fish in the northeast, such as carp, grass carp, and catfish. 

Furthermore, there was a significant upward trend in cities located along the middle and lower reaches of the Songhua River. Besides, the infection rate in Suihua has been more than 30% since 2010. This may be attribute to rich fish resources in the basin and local residents like eating raw fish. Thus, further prevention and control should been strengthened in these cities, especially promoting public health education among local residents [19]. No infection was found in the *Daxinganling* area. It may be that this area is located in the North of Heilongjiang Province, around the Greater Higgnan Mountains, with a long winter and short summer, and the average temperature is low. 

In this present study, men had a significantly higher egg-positive rate than women, which was consistent with other studies conducted in Vietnam, Korea and China [20–22]. Furthermore, the annual prevalence in men increased significantly. It is commonly assumed that this is due to the different way of life between men and women [23]. Men prefer to eat more fish and have more frequent social activities and eating opportunities at restaurants compared with women [14].

People of all ages are at risk and can be infected with *C. sinensis*. The prevalence rate increased gradually with age, reached a plateau among adolescents and young adults, and then decreased in elderly people. *C. sinensis* can survive for many years in the human body [1], therefore, this increase with age is considered to be the consequence of parasite accumulation. The decrease beyond the 70s is attributed to a higher death rate among infected people. Worryingly, the annual infection rate showed a significant increase in the 40–49 and 50–59 years groups. Therefore, they have become the main age groups that need prevention and treatment. 

The increased annual infection rate in rural areas was probably due to poor sanitary conditions, poor self-protection awareness, and lack of large-scale prevention education. This leads to a greater economic burden in rural populations. Furthermore, the infection rate among farm labourers was significantly higher than in the previous 3 years. Therefore, there seems to be a greater need for treatment, prevention and control of *C. sinensis* in rural areas. The infection rate of the civil servants in 4 years was increased, which might have been related to the rapid development of freshwater industries, the relatively slow detection and quarantine, development of fish food, and increase in staff eating out [24]. 

The strongest risk factor is thought to be the consumption of raw or uncooked freshwater fish and/or shrimps [2]. This habit has been prevalent in this endemic area for a long time. It is difficult to change this habit in the short-term for prevention and control, so the infection rate remains high. Otherwise, some people without a history of raw fish consumption were also found to be infected. It is probable that these people are infected by accidental ingestion of *C. sinensis* metacercariae from kitchen knives, towels and hands contaminated after catching and handling freshwater fish [25]. If food hygiene supervision is reinforced, infection of these populations could be reduced to some extent.

With regard to seasonality, individuals infected with *C. sinensis* were found all year. There is more opportunity to eat fish and shrimps in the summer. Although the rivers froze in the winter, many cases of infection were also found at that time. That is because it might take a period time to find the *C. sinensis* eggs or adults from initial infection to pathogenesis*.*

It is interesting to note that was some re-infection after treatment, as a result of further consumption of raw fish. Re-infection is still common in endemic areas and may be the main cause of consistent transmission [26]. The majority of people believe that clonorchiasis can be easily treated with oral praziquantel and will not result in serious hepatobiliary disease. So, re-infection is a major obstacle to control of clonorchiasis in endemic areas. In addition, the practice of home animal slaughter and poor sanitary conditions also increase the risk of re-infection [27]. Therefore, long-term follow-up and health education should be undertaken instead of a short period of patient evaluation in this group of patients [19]. 

Currently, praziquantel-based symptomatic chemotherapy is still the main treatment strategy [28]. However, it has not reduced the infection rate and prevented the increase in endemic areas. A multi-component integrated control programme like the integrated prevention and control strategies for schistosomiasis is required, such as avoiding eating raw fish, enhancement of health education programmes, and elimination of intermediate host snails [18,19].

This study had some limitations that need to be considered. First, the subjects were outpatients, which might have contributed to selection bias. This should be taken into account when extrapolating these findings to the general population in Heilongjiang Province. Second, this was a laboratory-based study, so exhaustive information related to patients’ clinical variables was unknown. Finally, we did not investigate the intermediate and reservoir hosts. 

In conclusion, the present study highlights that clonorchiasis remains a serious public health problem in Heilongjiang Province. Several risk factors have been shown to affect the prevalence, including eating raw freshwater fish and/or shellfish, sex, environment, and occupation. An integrated control programme including early diagnosis, medical intervention and promoting health awareness are imperative to reduce the public health impact of human clonorchiasis in this area of China. 
